# Omission of sentinel lymph node biopsy in breast cancer: a survey of Brazilian breast surgeons

**DOI:** 10.31744/einstein_journal/2026AO1673

**Published:** 2026-05-27

**Authors:** Erlan Clayton Xavier Cavalcante, Maria Clara Amorim Silva, Alana Maria Caland de Holanda Lustosa, Rafael Everton Assunção Ribeiro da Costa, Walberto Monteiro Neiva Eulálio, Cristiane Amaral dos Reis, Rodrigo José de Vasconcelos Valença, Elisa Rosa de Carvalho Gonçalves Nunes, Sabas Carlos Vieira

**Affiliations:** 1 Universidade Estadual do Piauí Health Science Center Teresina PI Brazil Health Science Center, Universidade Estadual do Piauí, Teresina, PI, Brazil.; 2 Universidade Federal do Piauí Health Science Center Teresina PI Brazil Health Science Center, Universidade Federal do Piauí, Teresina, PI, Brazil.; 3 Hospital sírio-libanês São Paulo SP Brazil Hospital sírio-libanês, São Paulo, SP, Brazil.; 4 Oncocenter Teresina PI Brazil Oncocenter, Teresina, PI, Brazil.

**Keywords:** Breast neoplasms, Mastectomy, segmental, Sentinel lymph node biopsy, Overtreatment, Surveys and questionnaires, Survival analysis

## Abstract

**Objective:**

Considering the increasing number of new breast cancer cases and the morbidity associated with axillary lymph node dissection in breast cancer treatment, this study evaluated the factors related to the omission of sentinel lymph node biopsy in early-stage breast cancer.

**Methods:**

A cross-sectional, observational, descriptive study was conducted using a digital questionnaire. Breast surgeons and physicians from other specialties currently practicing in Brazil were included. Data were tabulated and analyzed using Microsoft Office Excel®, version 2023. Statistical analyses were performed using JASP 0.19.1 software. Descriptive analysis was performed by calculating the absolute (n) and relative (%) frequencies of qualitative variables. Logistic regression analysis was performed using the stepwise method, and highly correlated variables were removed for the construction of the final model.

**Results:**

Of the 274 physicians, 158 (57.7%) reported omitting sentinel lymph node biopsy. Medical residency in surgical oncology, achievement of a specialist degree in mastology (TEMA - *Título de Especialista em Mastologia, é a especialidade mencionada*), time of practice in the specialty, sphere of professional practice, holding a Master's degree, and holding a doctoral degree were selected for multivariate analysis based on p-values and their greater clinical relevance. In the final logistic regression, statistical significance was observed for sphere of professional practice and holding a doctoral degree.

**Conclusion:**

The factors most strongly associated with omission of sentinel lymph node biopsy were working in the private sector and holding a doctoral degree.

## In Brief

New advances in the de-escalation of axillary surgery in patients with early-stage breast cancer have been in focus, specifically the omission of sentinel lymph node biopsies. Recent studies have reported similar survival outcomes and lower morbidity rates. However, this technique is not routinely used in clinical practice.

## Highlights

■ De-escalation of axillary surgery has become a priority in breast cancer treatment.
■ Factors associated with the decision to omit sentinel lymph node biopsy were collected via questionnaire.
■ Sentinel lymph node biopsy was omitted more often by private doctors and those holding a doctoral degree.
■ Continuing medical education initiatives are needed to update other groups.

## Graphical abstract

**Figure:**
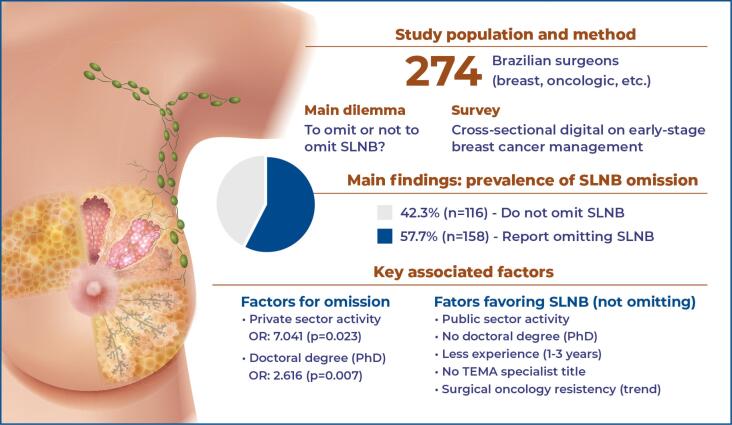

## INTRODUCTION

Sentinel lymph node biopsy (SLNB) is used to assess axillary involvement in patients with early-stage breast cancer and clinically node-negative axillae. In the late twentieth century, SLNB replaced axillary lymph node dissection after randomized trials demonstrated similar survival rates but lower morbidity.^(1,2)^

Over the last few years, new advances have resulted in the de-escalation of axillary surgery. A randomized clinical trial with a 10-year follow-up period (ACOSOG Z0011) evaluated the omission of axillary lymph node dissection in patients with early-stage breast cancer, clinically node-negative axillae, and up to two positive axillary sentinel lymph nodes undergoing breast-conserving treatment and radiation therapy and the study found similar overall survival and local recurrence rates between patients who underwent axillary dissection and those who did not.^(3)^

Therefore, SLNB may not confer significant benefits and, in some situations, may even increase associated morbidity. Moreover, reducing axillary interventions may improve patient quality of life and decrease treatment-related morbidity. In patients aged 70 years or older with luminal tumors and clinically node-negative axillae, the "Choosing Wisely" campaign recommends avoiding sentinel lymph node biopsy.^(4,5)^

Within this framework, the SOUND randomized study, published in 2023, demonstrated that omission of the SLNB procedure is non-inferior to SLNB in patients with early-stage breast cancer and clinically and ultrasonographically node-negative axillae. These results provide further support for SLNB de-escalation in breast cancer surgery, suggesting that patients with small tumors (diameter ≤2cm) and a negative result on preoperative ultrasound may safely avoid SLNB.^(6)^ Although the SOUND trial has had a significant impact on the decision-making of medical professionals, SLNB remains necessary for staging in many situations, such as in younger women (<50 years), patients undergoing mastectomy, those with human epidermal growth factor (HER2)-positive or triple-negative disease, or those undergoing neoadjuvant chemotherapy.^(6,7)^

Nevertheless, de-escalation of axillary surgery, including omission of SLNB in early-stage breast cancer, is not yet routinely performed in clinical practice by surgical specialists.^(8,9)^

Owing to the high prevalence of breast cancer and its impact on public health, characterizing how breast disease is managed by specialists in Brazil is essential. Advances in this area may significantly improve the quality of life of patients with breast cancer and help optimize public health costs. Therefore, the aim of this study was to

## OBJECTIVE

To analyze the factors associated with the breast surgeons’ decision-making regarding the omission of sentinel lymph node biopsy in Brazil.

## METHODS

### Study design

This was a cross-sectional, observational, descriptive study. Survey data were collected via an online digital form (Appendix A) using WhatsApp, Facebook, and face-to-face contact from May 2024 to July 2024.

### Study population

This study included breast surgeons and other specialties (general surgery, oncologic surgery, obstetrics and gynecology, and radiation therapy) currently practicing breast cancer surgery and living in Brazil. Non-medical professionals, those with incomplete or unanswered forms, and those who did not work in Brazil were excluded.

### Sample calculation

According to a survey conducted by the Brazilian Medical Association (AMB - *Associação Médica Brasileira*), there were 84655 physicians in 2022, including all the specialties considered in this study. In September 2024, the Brazilian Society of Breast Disorders (SBM) registered 1911 medical practitioners who were specialists in breast disorders: 1505 doctors with a Specialist Degree in Mastology (TEMA - *Título de Especialista em Mastologia, é a especialidade mencionada*) issued by the Brazilian Society of Breast Disorders and 406 doctors with a Specialty Qualification Record (RQE - *Registro de Qualificação de Especialidade*), issued by the Federal Medical Council (CFM - *Conselho Federal de Medicina*) for doctors who completed medical residency in breast disorders accredited by the Brazilian Ministry of Education (MEC - *Ministério da Educação do Brasil*) or obtained a specialist degree (TEMA).

The sample size was calculated considering a logistic regression model including six variables and an estimated event proportion of approximately 30%. This resulted in a required sample size of n=200, considering an infinite population, or n=182 when considering the number of doctors registered with the Brazilian Society of Breast Disorders. All responses received during the 8-week period were included, regardless of the minimum sample size.

### Variables

The following variables were analyzed: age of the professionals; year of graduation; medical residency completed (the study included general surgery, oncologic surgery, breast disorders, obstetrics and gynecology, and radiation therapy); having (or not) a specialist degree in breast disorders (TEMA); time of practice in current specialty; current sphere of professional practice (public and/or private); teaching activity; holding (or not) a Master's degree; holding (or not) a doctoral degree; use of a free informed consent form for omission of the SLNB; criterion used for the exclusion of the SLNB approach; technique used; number of cases performed with omission of the SLNB; and presence (or absence) of an axillary node only recurrence (in cases where SLNB was omitted).

### Statistical analysis

Data were tabulated and analyses were performed using Microsoft Office Excel^®^, version 2023 (Microsoft Corp., Redmond, NY, USA). Statistical analyses were performed using JASP 0.19.1 software program (https://jasp-stats.org/). Descriptive analysis was performed by calculating the absolute (n) and relative (%) frequencies of qualitative variables. Normality was measured using the Kolmogorov-Smirnov test. Because the entire sample showed a non-normal distribution, a univariate analysis was performed using the chi-square test. For multivariate analysis, variables with a p<0.05 and greater clinical relevance were selected, respecting the limit of six pre-established variables in the sample calculation. Logistic regression analysis was performed using the stepwise method, and highly correlated variables were removed from the final model.

### Ethical considerations

The study complied with all the Brazilian ethical principles for research (Resolution of the National Health Council N°466/12) and was approved by the Research Ethics Committee of the *Universidade Estadual do Piauí* (CEP/UESPI) (CAAE: 77671324.3.0000.5209; #6.793.712). By responding to the questionnaire, the doctors consented to participate in the research.

## RESULTS

In our study, of 274 doctors, 158 (57.7%) reported omitting SLNB, while 116 (42.3%) did not omit the procedure. The mean age of the participants was 48.6 years (46-50 years). The mean duration of practice in the specialty was 15.1 years. Medical residency in Breast Disorders was the most common residency, with 186 professionals (67.9%), while Radiation Therapy was the least frequent, with 0 (0%) professionals. Details of the study population and results of the univariate analysis are described in table 1.

**Table 1:** Distribution of factors related to the omission of sentinel lymph node biopsy: univariate analysis

		Do you omit the SLNB?				p value
		No		Yes		
		Count	% of total	Count	% of total	
Age (years)						
	26–30	1.0	0.365	1.000	0.365	0.189
	31–35	12.000	4.38	10.000	3.650	
	36–40	22.000	8.029	23.000	8.394	
	41–45	22.000	8.029	29.000	10.584	
	46–50	21.000	7.664	19.000	6.934	
	51–55	17.000	6.204	22.000	8.029	
	56–60	7.000	2.555	23.000	8.394	
	61–65	5.000	1.825	15.000	5.474	
	>65	9.000	3.285	16.000	5.389	
General surgery						
	No	74.000	27.007	113.000	41.241	0.175
	Yes	42.000	15.328	45.000	16.423	
Breast surgery						
	No	37.000	13.504	51.000	18.613	0.947
	Yes	79.000	28.382	107.000	39.051	
Surgical oncology						
	No	88.000	32.117	138.000	50.365	0.014
	Yes	28.000	10.219	20.000	7.299	
Obstetrics and gynecology						
	No	60.000	21.898	69.000	25.182	0.187
	Yes	56.000	20.438	89.000	32.482	
TEMA specialist degree						
	No	43.000	15.693	30.000	10.949	<0.001
	Yes	73.000	26.642	128.000	46.715	
Time of practice in the specialty (years)						
	1–3	12.000	4.380	8.000	2.920	0.012
	4–5	6.000	2.190	7.000	2.555	
	6–10	22.000	8.029	21.000	7.664	
	11–15	26.000	9.489	19.000	6.934	
	16–20	13.000	4.745	22.000	8.029	
	>20	37.000	13.504	81.000	29.562	
Sphere of professional practice						
	Public only	8.000	2.920	2.000	0.730	0.029
	Private only	23.000	8.394	42.000	15.328	
	Public and private	85.000	31.022	114.000	41.606	
Teaches						
	No	59.000	21.533	74.000	27.007	0.510
	Yes	57.000	20.803	84.000	30.657	
Master's degree						
	No	78.000	28.467	87.000	31.752	0.042
	Yes	38.000	13.869	71.000	25.912	
Doctoral degree						
	No	102.000	37.226	113.000	41.241	0.001
	Yes	14.000	5.109	45.000	16.423	
Uses a free informed consent form?						
	No	88.000	32.117	98.000	35.766	0.015
	Yes	28.000	10.219	60.000	21.898	
Exclusion criteria for SLNB approach						
Does not perform		116.000	42.336	0.000	0.000	<0.001
	>70 years and clinically node-negative and US node-negative axillae	0.000	0.000	100.000	39.496	
	>70 years and only clinically node-negative axillae	0.000	0.000	21.000	7.664	
	>60 years and clinically node-negative and US node-negative axillae	0.000	0.000	5.000	1.285	
	>50 years and clinically node-negative and US node-negative axillae	0.000	0.000	3.000	1.095	
	Any age and clinically node-negative and US node-negative axillae that will not alter the indication of systemic treatment	0.000	0.000	9.000	3.285	
	Others	0.000	0.000	20.000	7.299	
Which technique?						
	Does not perform	116.000	42.336	0.000	0.000	<0.001
	Patent blue dye	0.000	0.000	40.000	14.599	
	Technetium	0.000	0.000	39.000	14.324	
	Both	0.000	0.000	79.000	28.832	
How many cases have you operated on?						
	Never operated	98.000	35.767	0.000	0.000	<0.001
	Less than 10 cases	18.000	6.569	97.000	35.401	
	From 10 to 20 cases	0.000	0.000	37.000	13.504	
	From 21 to 30 cases	0.000	0.000	11.000	4.015	
	From 31 to 50 cases	0.000	0.000	6.000	2.190	
	More than 51 cases	0.000	0.000	7.000	2.920	
Did any of your patients have axillary node only recurrences?						
	No	0.000	0.000	154.000	96.855	0.872
	Yes	0.000	0.000	4.000	2.516	

Having completed medical residency in surgical oncology, having a specialist degree in breast disorders (TEMA), duration of practice in the specialty, sphere of professional practice, holding a Master's degree, holding a doctoral degree, use of the FICT, criteria used to exclude the approach, the technique used, and the number of cases operated without SLNB were considered significant factors (p<0.05) in the univariate analysis (Table 1). Based on p-values and greater clinical relevance, the following were selected for multivariate analysis: medical residency in surgical oncology, having a specialist degree in breast disorders (TEMA), duration of practice in the specialty, sphere of professional practice, holding a Master's degree, and holding a doctoral degree.

The final logistic regression model is described in table 2, where statistical significance was observed for the sphere of professional practice (specialists from the private sector had a greater tendency to omit SLNB) and for holding a doctoral degree. The remaining variables (medical residency in surgical oncology, having a specialist degree in breast disorders, duration of practice in the specialty, and holding a Master's degree) were excluded from the final model to avoid loss of accuracy and model significance.

**Table 2:** Logistic regression model: multivariate analysis

	Coeff.	p value	Odds ratio		
			OR	Lower boundary	Upper boundary
Omits SLNB					
Constant	-1.745	0.042			
Doctoral degree	0.961	0.007	2.616	0.264	1.659
TEMA	0.557	0.066	1.745	-0.037	1.151
Sphere of professional practice	1.952	0.023	7.041	0.264	3.640
Surgical oncology	-0.668	0.060	0.513	-1.365	0.028

OR: odds ratio; SLNB:, sentinel lymph node biopsy; TEMA:, specialist degree in breast surgery.

## DISCUSSION

The goal of surgical de-escalation via SLN biopsy omission is to reduce the morbidity associated with the surgical treatment of specific types of breast cancer, such as pain, lymphedema, and restriction of arm/shoulder mobility, all of which significantly impact the patients’ long-term quality of life.^(5)^

In this study, 158 practicing breast cancer surgeons (57.7%) reported omitting SLNB in patients with early-stage breast cancer. The literature suggests a slow adoption of updated protocols and guidelines for SLNB in several countries, which may explain the relatively balanced proportion of practitioners who omit SLNB and those who continue to perform it.^(10)^ Furthermore, although studies have shown that omission of SLNB does not adversely affect overall patient survival, its omission remains a concern to some specialists given that SLNB can influence systemic treatment decision-making and patient outcomes. This uncertainty can generate considerable apprehension among physicians regarding patient outcomes. Such concerns may partly explain the lack of a clear predominance of specialists who report omitting SLNB.^(9,11,12)^

In 2016, the Choosing Wisely campaign recommended against routine SLNB in patients aged 70 years or older with clinical stage I breast cancer, and those with luminal tumors, HER-2-receptor-negative disease, and clinically node-negative axillae, due to the lack of impact on oncological outcomes.^(8)^ The main study supporting this recommendation is the CALGB 9343 study; however, other current guidelines and recommendations also demonstrate that SLNB may not be necessary in patients older than 70 years of age and/or with poor performance status who have T1cN0 tumors that are HER2-negative and hormone receptor-positive, particularly when the indication for systemic treatment and/or radiation therapy would not be influenced by the result.^(13–16)^

In the current study, among the professionals who reported omitting SLNB, 100 (63.29%) used the following omission criteria: patients older than 70 years, those with HER2-negative luminal tumors, and those who were clinically and ultrasonographically node-negative; these criteria align with the descriptions in the literature. Axillary involvement in early-stage breast cancer is uncommon and may not justify SLNB given the potential morbidity associated with the procedure and the possibility that the risks outweigh the potential benefits.^(17)^

In addition, among the surgeons who reported the use of surgical de-escalation, the rate of axillary node-only recurrence was approximately 2.5%. Although this rate is not a determining factor in the decision-making process, it corroborates findings from clinical trials showing a low recurrence rate when SLNB is omitted, supporting the safety of this breast cancer management strategy. However, this rate may be susceptible to recall bias among practitioners who responded to the questionnaire, as these axillary recurrences could not be independently verified.^(18,19)^

Similarly, the European Oncology Institute reported no difference in overall survival among patients who did not undergo axillary surgery, and similar results were reported in another Swedish randomized trial.^(20,21)^ In contrast, a French study demonstrated poorer survival outcomes in patients who did not undergo axillary surgery. To explain these discordant findings compared to those of previous studies, the authors noted that chemotherapy was more frequently administered to the group that underwent axillary surgery. They emphasized that long-term follow-up is required to confirm or refute these initial observations.^(22)^

Moreover, data from The Surveillance, Epidemiology, and End Results (SEER) database were evaluated to determine the implementation rate of the Choosing Wisely recommendations in the United States. Multivariate analysis showed that the main factors associated with axillary surgery were Hispanic ethnicity, family income between $35,000 and $70,000, treatment provided in rural areas, undifferentiated tumors, lobular or mixed lobular-ductal histology, T2 tumors, and the use of radiation or systemic therapy. The median number of lymph nodes removed was two, with lymph node involvement occurring in only 2.1% of cases, highlighting the need for increased implementation of the Choosing Wisely recommendations in real-life clinical practice.^(23)^

In Brazil, the ongoing VENUS randomized study is evaluating the omission of SLNB in patients with early-stage breast cancer and clinically node-negative and ultrasound-negative axillae. This study includes patients with tumors measuring up to 5 cm, undergoing either breast-conserving surgery or mastectomy, with or without neoadjuvant chemotherapy, and is predicted to conclude in October 2025.^(24)^

Based on the results of this study and the available literature, the decision to omit SLNB appears to be multifactorial, considering both the patient status and characteristics of the disease, as well as the wide variations in the perspective of medical specialists regarding the performance of SLNB. For example, in our sample, 81 professionals (29.6%) with more than 20 years of medical experience reported omitting SLNB, whereas. only eight (2.9%) with 1-3 years of experience reported omitting SLNB. This finding suggests that longer practicing duration was associated with a greater tendency to omit SLNB, possibly reflecting increased confidence in clinical judgment developed over years of practice. This observation is consistent with studies indicating that both physician experience and patient preference can influence treatment decisions.^(12,25)^

In this context, factors related to higher levels of medical training may also influence the decision-making process. Among the respondents, 165 (60.2%) did not hold a Master's degree, and within this group, 87 (31.7%) reported omitting SLNB, and 78 (28.5%) did not. In contrast, of 109 (39.8%) respondents holding a Master's degree, 71 (25.9%) reported omitting SLNB, while only 38 (13.9%) did not, suggesting a greater tendency to omit SLNB among those with a higher academic training. Similarly, among the 59 (21.532%) respondents who held a doctoral degree, 45 (16.4%) reported omitting SLNB, whereas 14 (5.1%) did not. Therefore, omission of SLNB was more prevalent among those holding a doctoral degree than among those without one.

Variables such as holding a specialist degree in breast disorders (TEMA) and the sphere of professional practice, particularly working in the private sector, were also statistical significant factors influencing the decision-making process, possibly reflecting a greater level of professional updating among these physicians.

Although surgical de-escalation in breast cancer is a current and impactful topic, no large studies with a similar profile to ours, including consideration of the technical aspects of surgery and practitioner profile, have been conducted. The current study included a relatively larger sample size, which increases confidence in the findings. However, as the sample was not randomized, but instead obtained through professional groups and social media, selection bias cannot be ruled out; that is, specialists who omit SLNB may have been more likely to respond to the questionnaire. Therefore, further studies are warranted to better elucidate the factors associated with the omission of SLNB among Brazilian breast specialists. Such evidence could support the development of continuing medical education initiatives aimed specifically at younger specialists, those who do hold a doctoral degree, and those who work in the public sector.

## CONCLUSION

Among the Brazilian surgeons evaluated, a considerable proportion reported omitting SLNBs in patients with early-stage breast cancer. The factors most strongly associated with this practice included working in the private sector and holding a doctoral degree.

## Data Availability

The content is already available.

## Appendix A Questionnaire

1. Age of the doctor:
  - 21–25
  - 26–30
  - 31–35
  - 36–40
  - 41–45
  - 46–50
  - 51–55
  - 56–60
  - 61-65
  - +65
2. Graduation Year
3. Medical residency in:
  - General Surgery
  - Breast Disorders
  - Oncological Surgery
  - Radiation Therapy
  - Obstetrics and Gynecology
  - Others
4. If you marked "Others" above, specify which residency you completed.
5. Do you have a specialist degree in Breast Disorders? (TEMA)
  - Yes
  - No
6. How long have you been in practice?
  - 1–5 years
  - 6–10 years
  - 11–20 years
  - 21–30 years
  - +30 years
7. In which sphere of professional practice do you work?
  - Public only
  - Private only
  - Public and private
8. Are you a professor?
  - Yes
  - No
9. Do you hold a Master's degree?
  - Yes
  - No
10. Do you hold a doctoral degree?
  - Yes
  - No
11. Do you use a consent term for omission of the sentinel lymph node?
  - Yes
  - No
12. Criterion used for exclusion of SLNB investigation?
  - Patients aged > 70 years with luminal tumors, clinically node-negative and US node-negative axillae.
  - Patient over 70 years with luminal tumor and only clinically node-negative axillae.
  - Patients over 60 years of age with a luminal tumor, clinically node-negative and US node-negative axillae.
  - Patients older than 60 years with luminal tumors and only clinically node-negative axillae.
  - Patients over 50 years of age with luminal tumors and clinically node-negative and US node-negative axillae.
  - Patients over 50 years of age with luminal tumors and only clinically node-negative axillae.
  - Patients of any age with clinically node-negative and US node-negative axillae will not alter the indications for systemic treatment.
  - I do not omit the SLNB procedure
  - Others
13. In case of "Others", specify the criterion used:
14. Which technique was used?
  - Blue patent
  - Technetium
  - Blue patent and technetium
15. How many cases of invasive breast cancer have you operated on with the omission of SLNB investigation?
  - Less than 10 cases
  - From 10 to 20 cases
  - From 21 to 30 cases
  - From 31 to 50 cases
  - 51 or more cases
  - Never omitted the sentinel lymph node procedure
16. In case of omission of the SLNB procedure, have you ever experienced axillary node-only recurrence?
  - Yes
  - No
  - I do not omit the SLNB procedure
